# Analysis of spatial pattern and influencing factors of private clinics in the main urban area of Guiyang in China from 2021 to 2022 based on multi-source data

**DOI:** 10.1186/s13690-023-01068-5

**Published:** 2023-04-10

**Authors:** Wei Li, Fang-Juan Du, Ou Ruan

**Affiliations:** School of Geography and Environmental Sciences, School of Karst Science), Guizhou Normal University, Guiyang, 550001 Guizhou China

**Keywords:** Private clinics, Space pattern, Influence factors, Main urban area of Guiyang, Multisource data

## Abstract

**Background:**

Private clinics are important places for residents to obtain daily medical care. However, previous researches mainly focused on public medical institutions but ignored the issue of systematic allocation of social medical resources such as clinics. It is critical to understand the private clinics distribution to analyze the rational allocation of medical resources and the spatial difference.

**Methods:**

Based on the field survey, land census, population density, and economic data from Guiyang, this study analyzes the spatial pattern of private clinics in the main urban area of Guiyang and the influencing factors by using spatial analysis methods such as kernel density, standard deviation ellipses, and geo-detector.

**Results:**

The private clinics in the main urban area of Guiyang are characterized by "inner dense, outer sparse dense," showing an overall spatial clustering feature of "four cores and two belts with many points" and "dense inside and sparse outside." Different types of private clinics have distinct spatial distribution characteristics and agglomeration forms. The growth of private clinics is closely linked to the population growth of mountainous cities. The most important factors influencing the spatial pattern of private clinics are residential land factors, followed by traffic factors and population density. The impact of economic, natural, and spatial factors is minimal. When using a geo-detector, the results of multi-factor interaction differ from those of single factors, and factor interactions have greater explanatory power than single factors in clinic distribution.

**Conclusion:**

This study investigates the geographic distribution and influencing variables of private clinics in typical mountain cities and identifies the causes of the current disparity in the distribution of healthcare resources. It is necessary to gradually develop the primary healthcare system in mountainous cities with legislation, counterpart support, and social resources. While ensuring equal access to health care for low-income people and mobile populations, various medical needs of community members should be fully considered and implemented as soon as possible.

The issue of limited access to healthcare resources in China has received a lot of attention from researchers in a variety of fields [1]. One-fifth of the global total population of this country should be considered the need of medical resources, especially with the aging, and the rising prevalence of chronic non-communicable diseases in the next several decades. Therefore, the importance to the future development of China's healthcare is how to strengthen the network of community health service institutions, including both the public and also the private resources to meet the diverse needs of residents in terms of health services. Medical institutions that provide primary care are usually regarded as the foundation of the healthcare system, mostly consisted of clinics, township hospitals (health offices) in rural areas, and community health centers in urban areas in China [2]. Although the total number of existing medical and health institutions in China have reached 10,30,935 by the end of 2021, an increase of 8013 compared to 2020, with a growth rate of 0.78% [3], the location of primary healthcare facilities is crucial for offering individuals access to essential medical treatment in everyday life, which matters both the citizens' health and wellbeing, and the justice of healthcare.

Previous researches about the distribution of healthcare institutions focused on public institutions and the balance of healthcare resource allocation at various scales, mainly those state-owned ones [4–9], the descriptive analysis, the Lorenz curve, and the Gini coefficient are widely used to examine the equilibrium of medical resource allocation [10, 11], whereas geographic information technologies such as kernel density [12] and the two-step search method [13–15] are used to examine the spatial distribution and accessibility of the medical resources [16]. Obviously, these studies ignored the complementary role of private medical institutions, underestimated the function of their healthcare in China. Although a few studies have used point of interest data of private clinics into the primary care system, just only for a comprehensive analysis [17], nearly discussed the private clinics' distribution characteristics and influencing factors. With China's diversified medical development patterns, a large number of rapidly expanding private clinics have their characteristics and complement and interpenetrate with other medical systems, forming a ponderous system [18].

The environment for residents to access routine medical treatment has been improved by profit-driven clinics, especially in rural and urban areas that are still comparatively underdeveloped. However, private clinics are difficult to gain acceptance due to historical, governmental, and cognitive concerns. The "Guidance on Conducting Pilots to Promote Clinic Development" issued by the General Office of the National Health and Wellness Commission in 2019 further clarified the significance of clinics in implementing the Health China strategy and enhancing the overall effectiveness of medical services. Ten cities, including Beijing, Shenyang, Shanghai, Wuhan, Guangzhou, and Shenzhen, were taken as pilots to improve clinic construction and management policies [19].

Comparing with the state-owned hospitals, private clinics provide dental treatments [20], multi-morbidities, and easy-to-diagnose common disorders, with the flexible settings, useful services, a range of varieties, and reasonable prices [21, 22]. According to a large-scale national poll conducted in 2015, more than 80% of urban and rural inhabitants prefer community hospitals or clinics for the treatment of minor daily ailments [23], which could be shown that the private clinic is a typical primary medical institution and an essential part of the modern medical and health service system. The total number of clinics registered and operating in China increased from 259,833 in 2020 to 271,056 in 2021, accounting for 27.72% of the total number of primary healthcare institutions, with an increase of 4.32%, which far exceeds the overall growth rate of health care institutions [3]. It is the foundational that the spatial distribution of the private clinics is the complementary role in the primary care system.

Geographers are used to discuss the spatial distribution and analyze the geographical factors, including physical and social ones. This study focuses on the individual private profit-making medical institutions, private clinics, by using geographic probes for single factor and interaction analysis, especially as the case of study, Guiyang is a typical mountainous city, which means high input and low efficiency for public services. The goal of this paper is to give some reasonable suggestions for the spatial optimization of various medical resources, and further promote the medical resource to use and management effectively.

## Study area

**Fig. 1:**
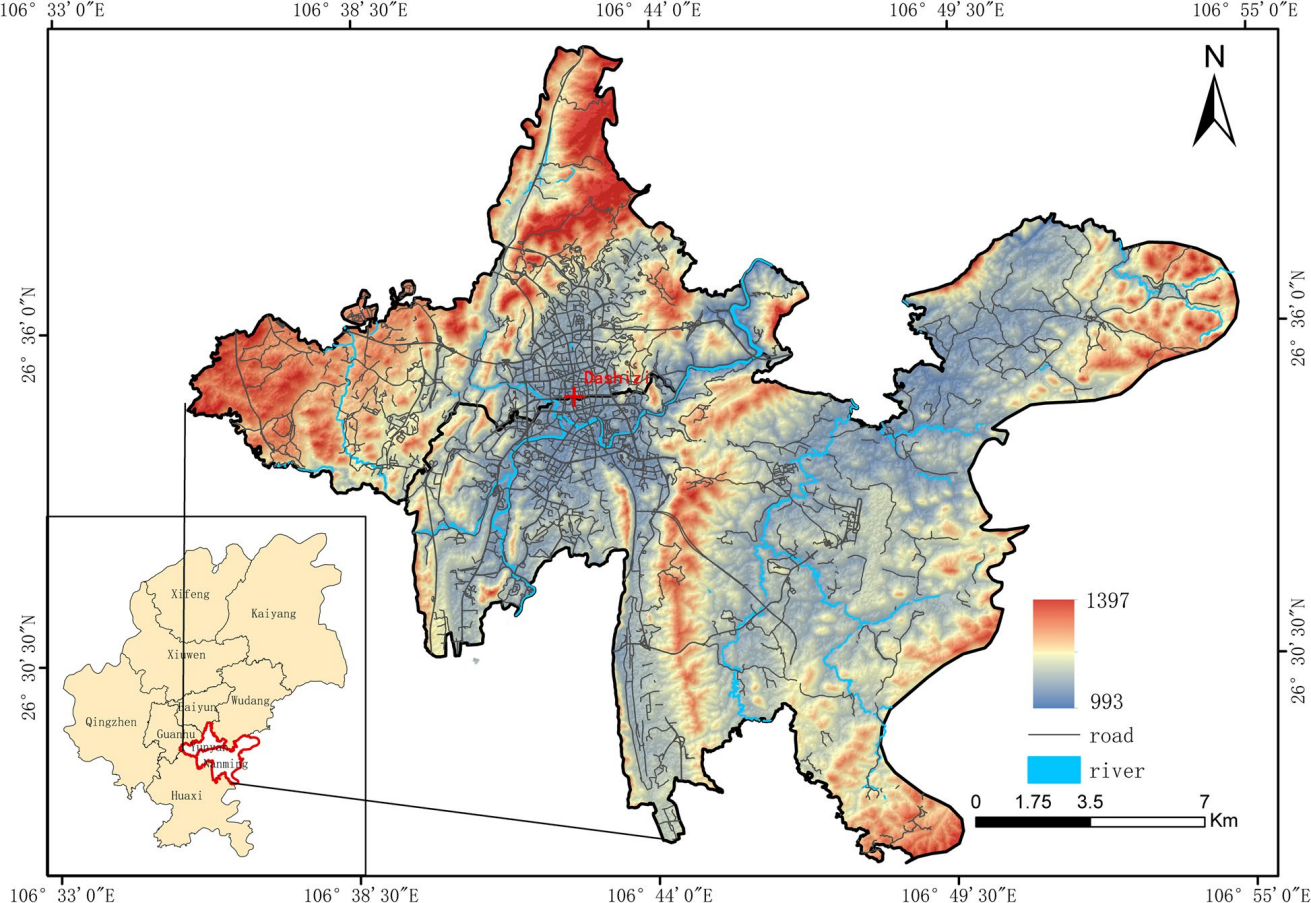
The study area of Guiyang

Guiyang, the capital of Guizhou Province, is located in the central part of the province. It is a typical mountainous city, and also an important transportation hub in the southwest region of China. Two urban areas of Guiyang City, i.e., Yunyan and Nanming, are located between 106°35′25′′-106°54′47 E and 26°27′05′′-26°40′39′′N, and cover an area of 303 km^2^, with 5 townships and 35 street offices and a resident population of 2.54 million. This research selects the central city of Guiyang as the study area, which has the longest history (Fig. 1). It shows both the evolution of modern urban development and the evolution of the pattern in administrative domination and urban renewal [24]. Simultaneously, all types of medical institutions are concentrated in this area, which has a strong distinctiveness.

## Data collection and research methods

### Data sources

①Basic maps and related data: the scope of the study area and administrative divisions were obtained from SkyMap thematic maps; the street population density data in 2020 were obtained from WorldPop (https://www.worldpop.org/), with a resolution of 1 km; the 1 km grid GDP data was obtained from the Chinese Academy of Sciences' Resource and Environment Science and Data Center; mountains, streets, and urban settlements were acquired via high-definition image interpretation based on field surveys. ②Data from the private clinic. According to the current situation of Guiyang city and based on the "Basic Standards for Clinics (Revised 2022)" and the related literature [25, 26], 716 valid medical service points were selected after six months of field survey according to the classification of medical and health service institutions by business nature, diagnosis and treatment subjects, and characteristic services (Table 1). The medical clinics were divided into five major categories: General clinic, traditional Chinese medicine clinic, integrated of Chinese traditional and modern medicine clinic, dental, and ethnic medical-clinic. This research did not include cosmetic and skin clinics, rehabilitation, ophthalmology, or psychiatric clinics. After the legitimacy of data from various sources was confirmed, a spatial database was created in ArcGIS, erroneous and aberrant data were deleted, and geographic coordinates were corrected.

**Table 1:** Classification of private clinics in main urban area of Guiyang in 2021–2022

Type		Number (pcs)	proportion(%)
Private clinics	General clinic	450	62.85
	Traditional Chinese medicine clinic	7	1.00
	Integrated Chinese and modern medicine clinic	87	12.15
	Dental	90	12.57
	Ethnic medical clinic	79	11.03
Total		716	100.00

### Research methods

The purpose of this paper is to: (1) use the kernel density estimation method to analyze the spatial clustering of private clinics in Guiyang City; (2) discuss the distribution centers and development trends of private clinics in Guiyang City using standard deviation ellipse pairs; (3) use the geographical detector to compare the explanatory power of multiple factors that contribute to the spatial pattern of clinics in Guiyang City and investigate the most influential factors.

#### Kernel density estimation method

The principle of the kernel density estimation method is based on clustering data density function calculation as a traditional method of exploring spatial density analysis. To calculate the density of the points, a circular area search is performed on each grid point [27]. The formulation of the kernel density function f(*x*) is shown below, which reflects the spatial location of *x* points.

1 $$\begin{array}{c}f_n\left(x\right)=\frac1{nh}\sum\limits_{i=1}^nk\left(\frac{x-x_i}h\right)\end{array}$$

where, ${f}_{n}\left(x\right)$ is the kernel density estimate of the center point of the private clinic service space in the study area; n is the number of samples in the center of the clinic service space (pcs); *h* is the area search radius (m); $x-{x}_{i}$ is the distance between two center points (m). The geometric meaning of this function is that the density value is highest at each center point, and it diminishes for the place far away from the center point. The larger the kernel density value, the higher the degree of aggregation.

#### Standard deviation ellipse

The spatial distribution direction and deviation degree of clinic services in the main urban area of Guiyang was investigated by comparing the diffusion direction and rotation angle of the standard deviation ellipse of different types of clinic services. The calculation formulas of this analytical method are as follows [28].

2 $$\begin{array}{c}{\sigma }_{x}=\sqrt{\frac{{\sum }_{i=1}^{n}({\omega }_{i}{\widetilde{x}}_{i}\mathit{cos}\theta -{\sum }_{i=1}^{n}{\omega }_{i}{\widetilde{y}}_{i}\mathit{sin}\theta {)}^{2}}{{\sum }_{i=1}^{n}{{\omega }_{i}}^{2}}}\end{array}$$

3 $$\begin{array}{c}{\sigma }_{y}=\sqrt{\frac{{\sum }_{i=1}^{n}({\omega }_{i}{\widetilde{x}}_{i}\mathit{sin}\theta -{\sum }_{i=1}^{n}{\omega }_{i}{\widetilde{y}}_{i}\mathit{cos}\theta {)}^{2}}{{\sum }_{i=1}^{n}{{\omega }_{i}}^{2}}}\end{array}$$

4 $$(\stackrel{-}{x, }\overline{y }=\left[\frac{{\sum }_{i=1}^{n}{\omega }_{i}{x}_{i}}{{\sum }_{i=1}^{n}{\omega }_{i}},\frac{{\sum }_{i=1}^{n}{\omega }_{i}{y}_{i}}{{\sum }_{i=1}^{n}{\omega }_{i}}\right]$$

5 $$\begin{array}{c}tan\theta =\frac{\left(\left({\sum }_{i=1}^{n}{\omega }_{i}^{2}{\widetilde{x}}_{i}^{2}-{\sum }_{i=1}^{n}{\omega }_{i}^{2}{\widetilde{y}}_{i}^{2}\right)+\sqrt{{\left({\sum }_{i=1}^{n}{\omega }_{i}^{2}{\widetilde{x}}_{i}^{2}-{\sum }_{i=1}^{n}{\omega }_{i}^{2}{\widetilde{y}}_{i}^{2}\right)}^{2}-4{\sum }_{i=1}^{n}{\omega }_{i}^{2}{\widetilde{x}}_{i}^{2}{\widetilde{y}}_{i}^{2}}\right)}{2{\sum }_{i=1}^{n}{\omega }_{i}^{2}{\widetilde{x}}_{i}{\widetilde{y}}_{i}}\end{array}$$

where ${x}_{i},{\gamma }_{i}$ is the center coordinate of each cell in the study area; $\stackrel{-}{x, }\overline{y }$ is the center of gravity coordinate; $\omega_{i}$ is the weight of the cell; $\theta$ is the azimuth of the ellipse; $\widetilde{x}_{i} ,\widetilde{y}_{i}$ is the deviation of the center coordinate of each cell from the center of gravity; $\sigma_{x} ,\sigma_{y}$ is the standard deviation along the $x$ axis and $x$ axis, respectively.

#### Geo-detector

Geo-detector is a set of statistical methods for detecting spatial heterogeneity and revealing the influence factors. It is good at analyzing type quantities, both numerical and qualitative data, as well as detecting the interaction of two factors on the dependent variable and testing its statistical significance [29].

##### Single influence factor analysis

In this research, the factor detection module is primarily used to introduce the spatial distribution determination force indicator q for the private clinics in the main urban area of Guiyang City. The formula for calculating *q* is shown below.

6 $$q=1-\frac{\sum_{h=1}^{L}{N}_{h}{\sigma }_{n}^{2}}{N{\sigma }^{2}}=1-\frac{SSW}{SST},SSW=\sum_{\mathrm{h}=1}^{\mathrm{L}}{\mathrm{N}}_{\mathrm{h}}{\upsigma }_{\mathrm{h}}^{2},SST=\mathrm{N}{\upsigma }^{2}$$

Where, *h* = 1,2…, *L* is the number of strata of the influence factor *X* on the spatial distribution of clinics; *Nh* and *N* are the number of cells within stratum *h* and the whole study area, respectively; ${\sigma }_{h}^{2}$ and $\sigma$ are the variances of *Y* values in stratum *h* and the whole study area, respectively; *SSW* and *SST* are the sums of the stratum variance (within the Sum of squares) and the total variance of the whole area (total sum of squares) [29]. *q* falls within [0, 1], and a larger value of *q* indicates that the factor has a greater influence on the distribution in the clinic space and vice versa. *q* = 1 means that the independent variable *X* completely controls the variance of clinic space; *q* = 0 means that the spatial pattern of clinics is randomly distributed and the independent variable *X* has no effect on the variance of private clinic space.

##### Influence factor interaction analysis

The interaction detection module is used to investigate the interaction between the spatially distributed independent variable factors in the clinic, and the q-values of the respective variable factors, as well as the strength of their explanatory power on the dependent variable Y are compared. The q-value magnitudes of factors X1 and X2 on Y, i.e., q(X1) and q(X2), and the q-value magnitudes when X1 and X2 interact, i.e., q(X1 X2), are calculated using the above equation. The interaction types of q(X1), q(X2), and q(X1 X2) are compared, and the interaction types between the two factors in the private clinic distribution can be divided into the following categories (Table 2).

**Table 2:** Type of two-factor interaction results

Condition	Type of interaction
q(X_1_ ∩ X_2_) < Min(q(X_1_),q(X_2_))	Non-linear weakening
Min(q(X_1_),q(X_2_)) < q(X_1_ ∩ X_2_) < Max(q(X_1_),q(X_2_))	Single-factor nonlinear attenuation
q(X_1_ ∩ X_2_) > Max(q(X_1_),q(X_2_))	Two-factor enhancement
q(X_1_ ∩ X_2_) q(X_1_) + q(X_2_)	Independent role
q(X_1_ ∩ X_2_) > q(X_1_) + q(X_2_)	Non-linear enhancement

##### Selection of influencing factors

Most of the existing studies on the influencing factors of medical resource allocation use qualitative descriptions [30, 31] or investigate the drivers of medical resource distribution through the weighted regression models and superposition analysis [32, 33]. However, there are few quantitative studies for each driver, which fail to reveal the spatial structural characteristics of medical resources and the impact of their influencing factors. Based on the distribution range and pattern of clinics in Guiyang's main urban area, this study exploits available and reliable multi-source spatial data and refers to previous studies on the spatial analysis of medical resources such as public medical care and tertiary hospitals in the city [12, 17], as well as the "spillover" effect of public medical institutions such as hospitals (Table 3). The spatial distribution of clinics in Guiyang's main urban area was modeled by using eight measurable proxies in five dimensions, including natural environmental factors, demographic factors, transportation conditions, economic development level, and spatial distance.

**Table 3:** The evaluation indexes of private clinics in the main urban area of Guiyang

Dimension	Proxy variable	Data sources and calculation methods (unit)
**Natural environment**	***X*1** DEM	Extracted by GIS analysis (m)
	***X*2** SOLP	Extracted by GIS analysis (%)
**Economic**	**X3** GDP per capita	Center for Resources and Environmental Science and Data, Cas(1 km × 1 km)
**Demographic**	***X*4** Population density	WorldPop (2020 https://www.worldpop.org/)(person/km^2^)
**Traffic**	***X*5** Road density	HD satellite image interpretation(km/km^2^)
**Land-use**	***X*6** Share of residential land area	HD satellite image interpretation(km^2^)
	***X*7** Share of commercial land area	HD satellite image interpretation(km^2^)
**Spatial distance**	***X*8** Distance to hospitals above the municipal level	Extracted by GIS analysis(km)

In this study, the main urban area of Guiyang City was created based on the field survey. Grid coordinate points (1 km × 1 km) were collected in the fishnet, and the grid was used taken the research unit, while the data-free area was excluded to avoid the influence of different administrative divisions on the analysis results. Following Jenks' natural best breakpoint method, the factors were classified into five levels, and the spatial distribution of the clinic services in the main urban area of Guiyang was analyzed through factor detection and interaction detection using geographic probes. Then, the determining power and significance level of each factor on the spatial distribution pattern of clinics were derived, and the types of interaction between the factors of each variable were compared.

## Results

### Distribution characteristics of private clinics in the main urban area of Guiyang

#### General distribution pattern of private clinics

**Fig. 2:**
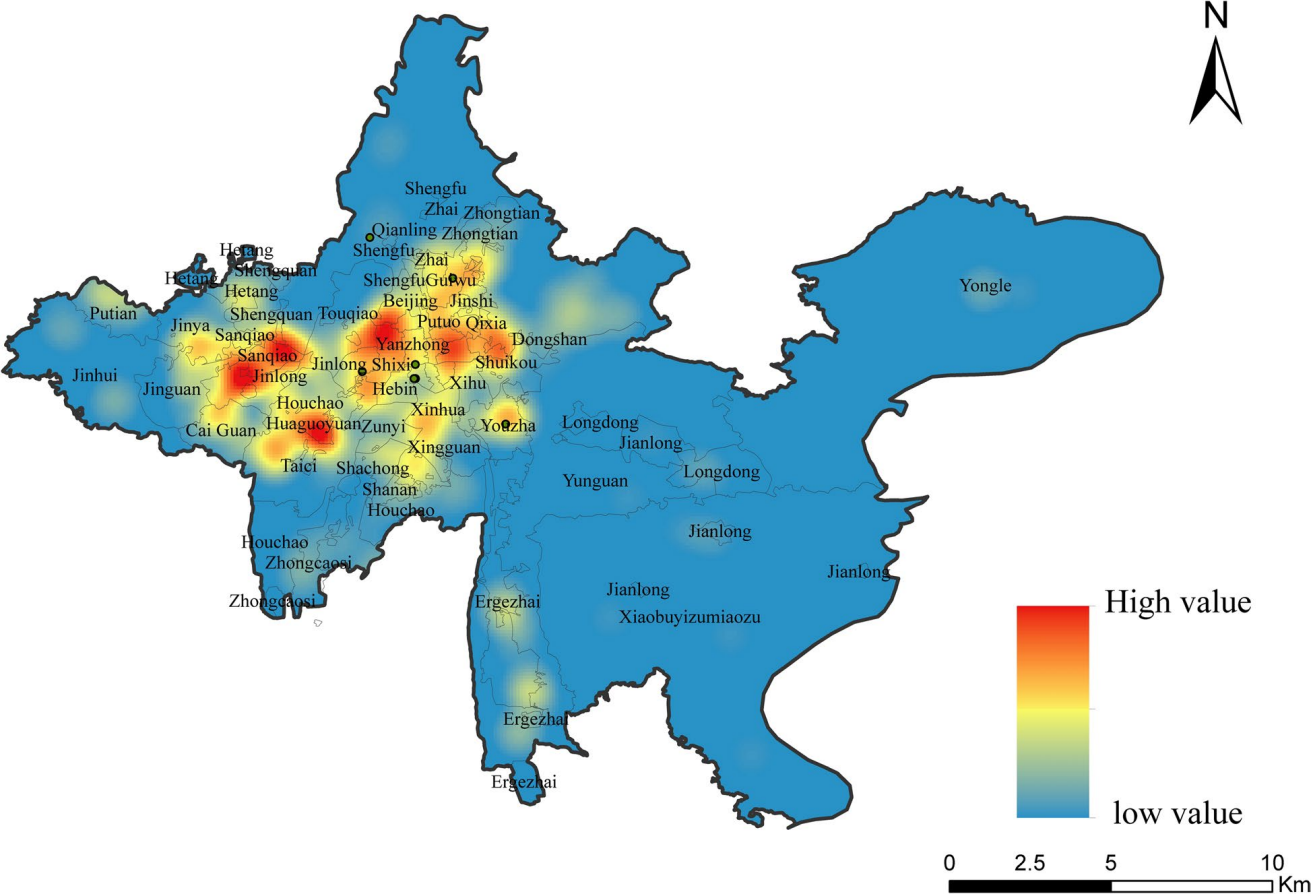
The spatial pattern of private clinics in main urban area of Guiyang in 2021–2022

The kernel density analysis method was exploited to obtain the overall pattern of private clinic distribution in Guiyang's main urban area, and the kernel density distribution map was created using ArcGIS 10.6 (Fig. 2). It can be seen that clinics in Guiyang's main urban area are generally unevenly distributed in the west and east, forming a pattern of "four cores, two belts, and multiple points," which is consistent with Guiyang's urban layout. The "four cores" have the highest distribution density and are located in Guiyang's central city and suburbs. The central city consists of old neighborhoods like City West, Dongshan, Yanzhong, and Weiqing, while the suburbs consist of old industrial areas like Sanqiao, Jinlong, Hetang, and Putian, as well as large developments like Huaguoyuan. The "two belts" are mostly areas radiating from the city center, such as the communities of Zhaiji and Guiwu in the north and Xingguan and Shannan in the south. The "multiple points" are scattered throughout the core areas of each community and township.

**Fig. 3:**
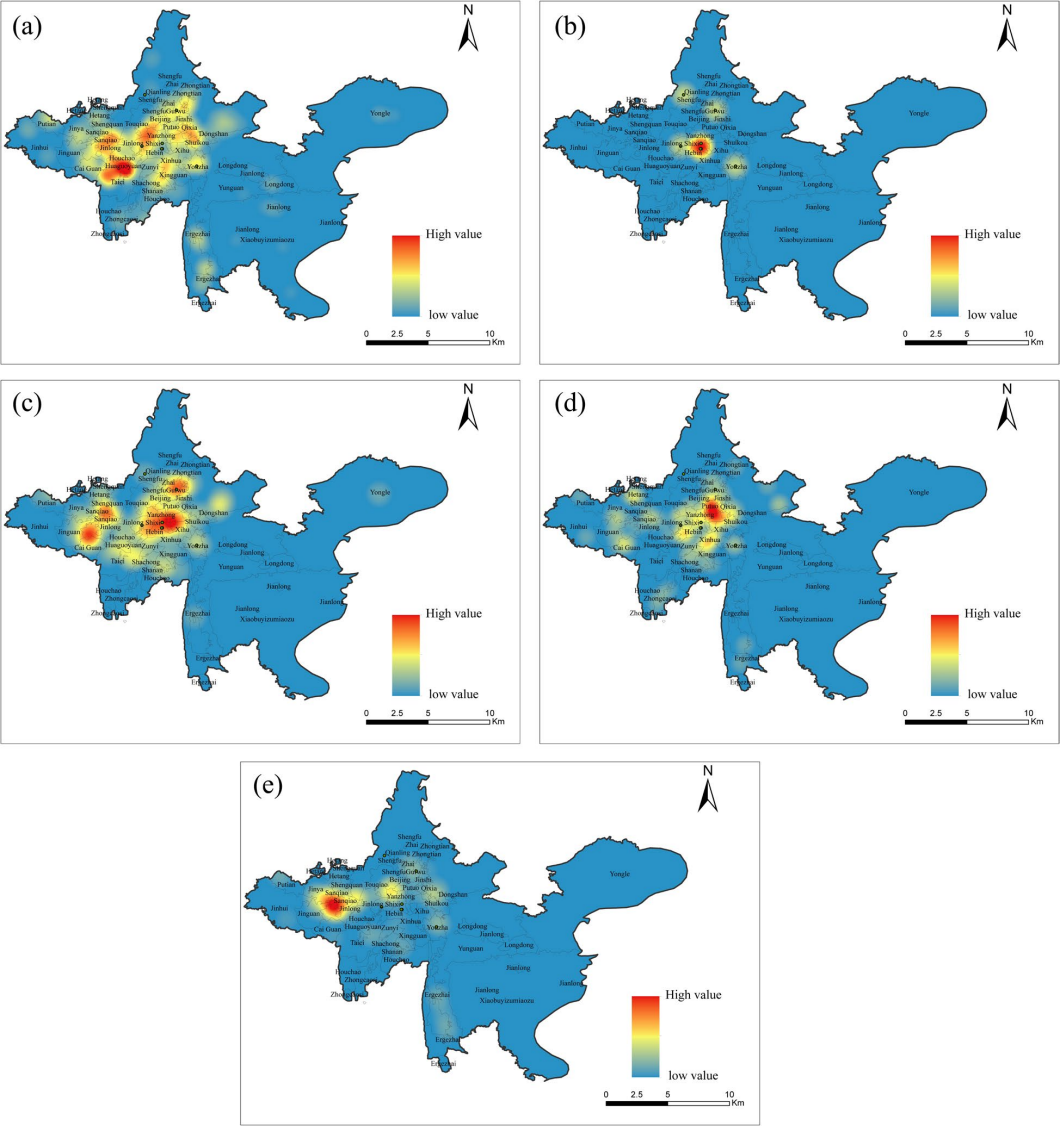
The kernel density map of various private clinics in the main urban area of Guiyang in 2021–2022. **a**. General clinic; **b**. traditional Chinese medicine clinic; **c**. Integrated of Chinese and modern medicine clinic; **d**. dental; **e**. ethnic medical clinic)

Combined with Fig. 3, it can be seen that the spatial distribution of various types of private clinics in Guiyang City exhibits distinct distribution patterns of polycentric clustering. General clinic and integrated of Chinese and western medicine clinics are distributed in the main urban area of Guiyang city in a large range of "surface + point". The multiple cores cluster together to form a large scale cluster area, with a wide distribution and influence range, and the core areas of the two clinics are slightly different. The distribution of Chinese clinic, dental clinics, and ethnic medical clinics exhibit a pattern of "one core multi-point," but the core locations differ significantly, with the former two types of clinics concentrated in old urban areas and the latter in suburban interface and old industrial areas.

#### Spatial distribution direction of private clinics

**Fig. 4:**
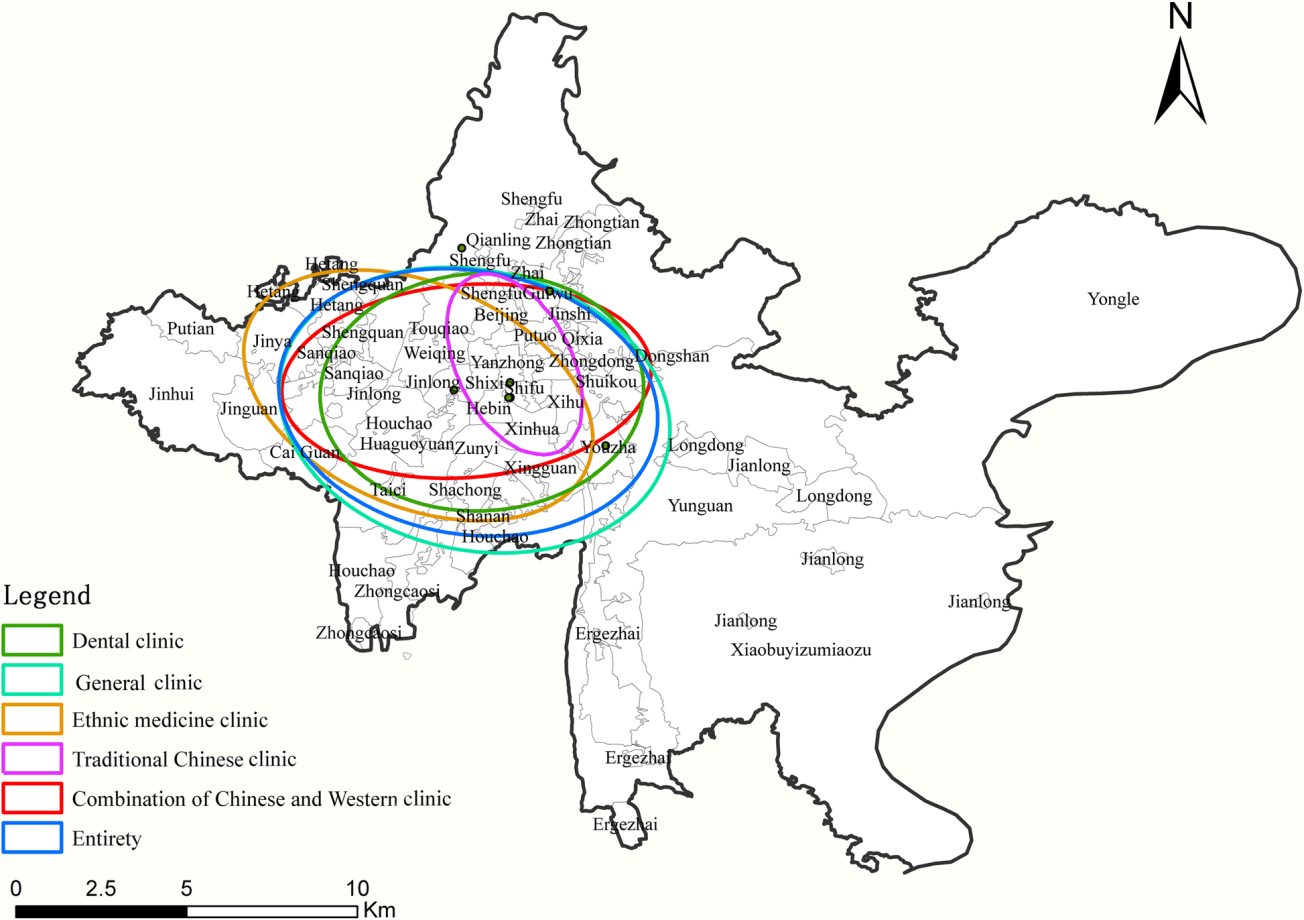
The standard deviation ellipse diagram of various private clinics in main urban area of Guiyang in 2021–2022

The spatial layout of private clinics in main urban area of Guiyang City was further investigated by using the standard deviation ellipse analysis method. The results that the overall standard deviation in the ellipse’s long-axis direction of the clinic space is 13.20° to the south of the east, indicating an eastward-westward pattern. Except for TCM and ethnomedicine, the standard ellipse of all types of clinic service spaces overlaps; the ellipse centers of the other types of clinics are close to each other and parallel to the overall distribution direction. Meanwhile, the ellipse centers of the traditional treatment places like TCM and ethnomedicine are westward, and the long-axis direction of the standard deviation ellipse is 62.49° and 28.50° to the west of the north, respectively (Fig. 4). On the one hand, the majority of the mountains straddling Guiyang's central city have a north–south orientation, which hampers the city's early east–west expansion, and the city mainly extends longitudinally along with the orientation of the mountains. On the other hand, the distribution of medical services is related to the direction of urban expansion [27], functional positioning, and infrastructure construction [34].

### Study on the influence mechanism of the spatial distribution of private clinics in the main urban area of Guiyang

#### Single-factor results for the spatial distribution of private clinics

The collected sample information was imported into the model, and the results are shown in Table 4. It can be seen that the eight influencing factors had significant explanatory power for the spatial heterogeneity of the clinics in the main urban area of Guiyang, but the explanatory power and significance of different influencing factors were different.

**Table 4:** The explanatory power of the impact factors on the distribution of private clinics in main urban area of Guiyang in 2021–2022

	***X1***	***X2***	***X3***	***X4***	***X5***	***X6***	***X7***	***X8***
clinic	0.0519**	0.0198	0.2328***	0.2507***	0.3883***	0.5307***	0.1122***	0.0504**

Dependent variable: * *p* < 0.10, ** *p* < 0.05, *** *p* < 0.001

Five of the eight influencing factors for clinic distribution passed the significance level test of 0.001, including GDP per capita, population density, road density, the share of residential land area, and commercial land area. Meanwhile, two factors, namely the elevation, and distance to hospitals above the municipal level, passed the significance level test of 0.05. For the spatial distribution of private clinics in the main urban area of Guiyang, residential land had the highest explanatory power of 0.5307, followed by road density (0.3883), population (0.2507), and GDP (0.2328), while commercial land (0.1122), DEM (0.0519), and distance to hospitals above the municipal level (0.0504) had modest explanatory power for clinic distribution, although slope has no significant influence.

#### Results of the interaction of factors influencing the spatial distribution of private clinics

The interaction detection module of the detector was used to obtain the q-values of the interaction of the influencing factors, and the results are shown in Table 5. It can be seen that the q-values of the interaction of the independent variable factors had greater explanatory power than a single factor in influencing the spatial clinic distribution, indicating that the spatial distribution of private clinics is affected by multiple factors. By comparing and ranking the influencing factors with greater explanatory power, the interactive driving factors influencing the spatial distribution of private clinics were: share of residential land area ∩ slope (0.6196), share of residential land area ∩ road density (0.5910), share of commercial land area ∩ road density (0.5901), share of residential land area ∩ elevation ( 0.5874), residential land area ratio ∩ population density (0.5843), residential land area ratio ∩ GDP per capita (0.5617), share of residential land area ∩ distance to hospitals above municipal level (0.5354), population density ∩ road density (0.4638), road density ∩ slope (0.4553), and road density ∩ GDP per capita ( 0.4532).

**Table 5:** The interaction detection results of factors influencing the spatial distribution of private clinics in the main urban area of Guiyang in 2021–2022

	***X1***	***X2***	***X3***	***X4***	***X5***	***X6***	***X7***	***X8***
*X1*	0.0519							
*X2*	0.1039	0.0198						
*X3*	0.3183	0.2955	0.2328					
*X4*	0.3527	0.3936	0.3546	0.2507				
*X5*	0.4375	0.4553	0.4532	0.4638	0.3883			
*X6*	0.5874	0.6196	0.5617	0.5843	0.5910	0.5307		
*X7*	0.3026	0.1972	0.3680	0.4090	0.4954	0.5901	0.1122	
*X8*	0.1344	0.0897	0.2558	0.2579	0.4023	0.5354	0.1677	0.0504

Besides, among the interaction detection results of each influencing factor, the results of distance to municipal hospitals ∩ DEM, distance to municipal hospitals ∩ slope, DEM ∩ slope, DEM ∩ share of commercial land area, DEM ∩ population density, DEM ∩ GDP, share of residential land area ∩ slope, slope ∩ share of commercial land area, slope ∩ population density, slope ∩ road density, slope ∩ GDP, share of commercial land area ∩ population, and share of commercial land area ∩ GDP results were nonlinearly enhanced ([q(X1 ∩ X2) > q(X1) + q(X2)]), and the interactions among the remaining influencing factors were bivariate enhanced {q(X1 ∩ X2) > Max[q(X1),q(X2)]}.

## Discussion

According to the calculation of the above influence, the distribution of private clinics in the main urban area of Guiyang has obvious spatial differentiation and clustering characteristics. Also, the clinic distribution is affected by urban development processes, socioeconomic and policy factors, etc.

### Analysis of the influence factors of clinic distribution in the main urban area of Guiyang City based on geo-detectors

#### Analysis of a single-factor model detection

The main factors influencing the spatial distribution of clinics are residential land use, road density, population density, and GDP per capita, while factors such as commercial land use, elevation, slope, and distance to hospitals above the municipal level have a weak influence. ① Residential land use is the primary factor influencing the spatial distribution of private clinics in Guiyang. It indicates that the medical and health services should be sufficiently close to the service recipients, i.e., the source of the recipients of medical care has a fundamental influence on the spatial distribution of medical care. This also indicates that clinics, as a medical professional field, have developed into the living service industry and become an important place for providing daily medical services [35], and the observations are consistent with national statistics [3]. ② Residents rely on transportation road networks to access these services, such as seeking medical advice and purchasing medicines. The greater the density of the road network, the better the traffic accessibility, and the denser the clinic outlets [17]. This is more prevalent in cities with mountains. The degree of medical resource distribution and aggregation is closely related to the accessibility of metropolitan highways. ③ The areas with high road density are mostly located in areas of higher economic development, and thus have a higher population and GDP per capita, which fully confirms the previous analysis results of the nuclear density pattern of clinic distribution. It demonstrates that the population flow brought about by the dense distribution of residential neighborhoods and roads gives life to the clinic service space. Meanwhile, the clinic space has more advantages than public medical facilities in terms of distance [36]. It can also acquire more service groups and a larger service radius with the help of densely populated neighborhoods and an established traffic network, contributing to better economic effects.

#### An examination of the factor interaction model

The interactions between the influencing factors are all two-factor enhanced or non-linearly enhanced, and multiple factors have significantly greater explanatory power than a single factor. The findings suggest that the spatial distribution pattern of private clinics in the main urban area of Guiyang is the result of a multi-factor interaction with augmentation. ①When interacting with other factors, the area share of residential land has the strongest the highest explanatory power when interacting with slope. The interaction between commercial land use and road density reveals the clinic's proximity characteristics as a profitable medical site and the distribution of service recipients. ② Topography was considered an important local factor in the spatial allocation of medical resources [12], but in the one-factor detection model, the slope had no significant effect on the clinic distribution in Guiyang, a typical mountainous city. Though the DEM had some explanatory power, it was not a major influencing factor. The relationship between natural elements in mountainous cities and settlement dispersion, road density, and other characteristics can be explained by the interaction of influencing factors. The data indirectly show how topography influences the structure of mountain cities and the geographical distribution of medical services.

### The impact of urban development patterns on the spatial distribution of clinics

The long axis of the standard deviation ellipse of general clinics, integrated Chinese and western medicine clinics, and dental clinics shows an east–west distribution, whereas the long axis of the ellipse of Chinese medicine and ethnic medicine shows a significant north–south distribution. The inherent geography of mountainous cities suggests that the pattern of cities tends to be from a single core to several satellite cities [37]. The same is true of Guiyang. With the development of transportation corridors like roads, bridges, and tunnels, Guiyang gradually overcame the mountain barrier and expanded outward. These transportation corridors are objective influencing factors of the unequal distribution of urban and rural medical resources. Guiyang was originally a relatively flat basin. Quality medical resources have long been concentrated in the central city and have not been effectively relocated and supplemented with urban expansion and population growth, which have created an opportunity for private practice development.

### The relationship between policy development and the spatial distribution of health care resources

The distribution of clinics has a clear preference for residential land, densely populated areas, and densely populated areas. The implementation of relevant policies by the Chinese government has played a significant role in guiding the evolution of urban geographic functions and the allocation of various resources. The implementation of policies governing clinic approval has promoted the rapid development of social medicine and health services. Meanwhile, since 2010, Guiyang has been constructing a large number of commercial and subsidized housing units in the west, which has attracted a certain amount of foreign and mobile population, and the focus of urban development has gradually shifted from the old city to the west [38]. In response to urbanization, the government is actively strengthening medical resource integration and implementing a hierarchical diagnosis and treatment system, as well as gradually improving medical service accessibility and coverage. However, large public hospitals in Guiyang are overcrowded due to finite high-quality resources. This results in an objective "mismatch" between population distribution and quality medical resources, and it makes blank space for the growth of informal ethnic medicine. In terms of spatial structure, the distribution of clinics in Guiyang's main urban area exhibits significant spatial differentiation and clustering characteristics, indicating that the central urban area and the suburban junction are more balanced, while the townships are sparser. Meanwhile, this study discovered that some townships around Guiyang failed to form effective detection data points due to a lack of medical resources. It shows that medical scarcity zones of public medical resources at various levels are easy to establish in remote cities and localities, which is more typical in China's western region [39]. According to the survey findings, social capital is difficult to concentrate in these areas due to the absence of economic development dynamics, and public healthcare resources are required to satisfy people's fundamental healthcare needs [12].

### Interaction between public health services and healthcare-seeking behavior

The assurance of high-quality urban life, which is related to social and spatial justice, is provided by reasonable matching and effective utilization of medical resources in space [40, 41]. The fundamental difference of the type of health systems in China is the dual medical systems of indigenous medicine and Western or modern medicine in contrast to developed countries and regions [42]. Evidence from some developing countries, shows that traditional medical practitioners-including herbalists, diviners and others—form the main body of primary health workers in many rural areas, and even urban populations rely on and patronize traditional health systems [43, 44]. A considerable part of Guiyang's medical and health resources are provided by private clinics, which is tied to the market demand created by locals' regular medical practices. The majority of the 80 informal ethnic medical sites discovered during this survey are located in shantytowns, abandoned industrial zones, or next to farmers' markets in protected housing and demolition and resettlement sites at the border of suburbs, where a disproportionately large percentage of impoverished and migrant residents live together with public healthcare facilities of relatively low quality. Once the residents are ill, they will select private clinics because they are quick, affordable, and convenient, creating a niche market for illegal or ethnic private medical care. In informal medical settings, the hygienic environment and technical proficiency of the medical staff are usually not guaranteed [45], and there are higher medical hazards. These residents' medical conduct and social traits will have a big impact on their health in the long run [46]. Generally, urban low-income persons and the mobile population experience varying degrees of difficulty in obtaining medical consultation clinics due to low levels of health awareness, off-site medical policies, reimbursement bases, and insufficient consumption of health services [47, 48].

## Conclusion

According to the field work and analysis, we found that there are 716 private medical clinics of five major categories, among which the major part is the general clinics, and traditional Chinese medicine clinics account for only 1%, less than integrated Chinese and modern medicine clinic. By contrast, the ethnic medical clinics are unexpectedly above 10%, which is noticeable that the alternative role of health service for the target group, and the supervision and management to the local medical administrations. It is important to promote the coordinated development of various types of medical and health services, and improve the health and well-being of all people.

The distribution of these private clinics has obvious spatial differentiation and clustering characteristics, especially distributed unevenly in the west and east of Guiyang's main urban area, the "four cores" have the highest distribution density and are located in Guiyang's central city and suburbs. It is necessary to consider how to balance the resource allocation between urban and suburban district areas and provide quality healthcare services to people of all various social classes.

The main factors influencing the spatial distribution of clinics are residential land use, road density, population density, and GDP per capita. which shows that social healthcare resources could be as a spot to understand the whole of the healthcare system of less-developed western mountainous cities in China'. Comparing with the plain cities, the medical resource allocation guidelines in mountain cities must consider the topographic characteristics, residential land use, population density in the difference of administrative regions [7]. As same as the assistance of legislation, counterpart assistance, social resource utilization etc..

## Data Availability

The datasets used during the current study available from the corresponding author on reasonable request.
